# Supplementary figures and images for: Detection of SARs-CoV-2 in wastewater using the existing environmental surveillance network: A potential supplementary system for monitoring COVID-19 transmission
Source: PLoS One. 2021 Jun 29;16(6):e0249568. doi: 10.1371/journal.pone.0249568 (PMC8241060; doi:10.1371/journal.pone.0249568)

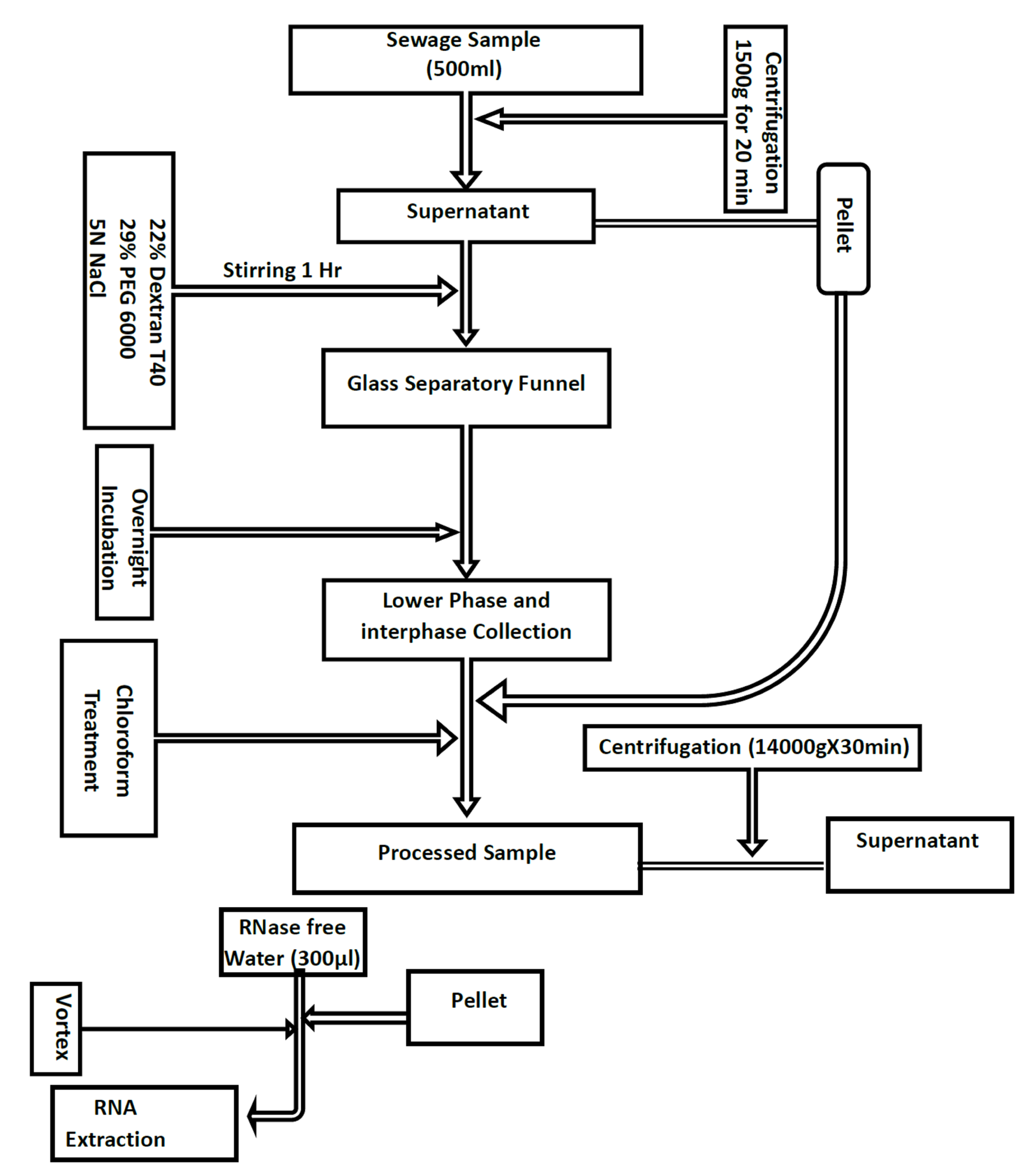

Supplement: S1 Fig — (TIF) [file pone.0249568.s001.tif]

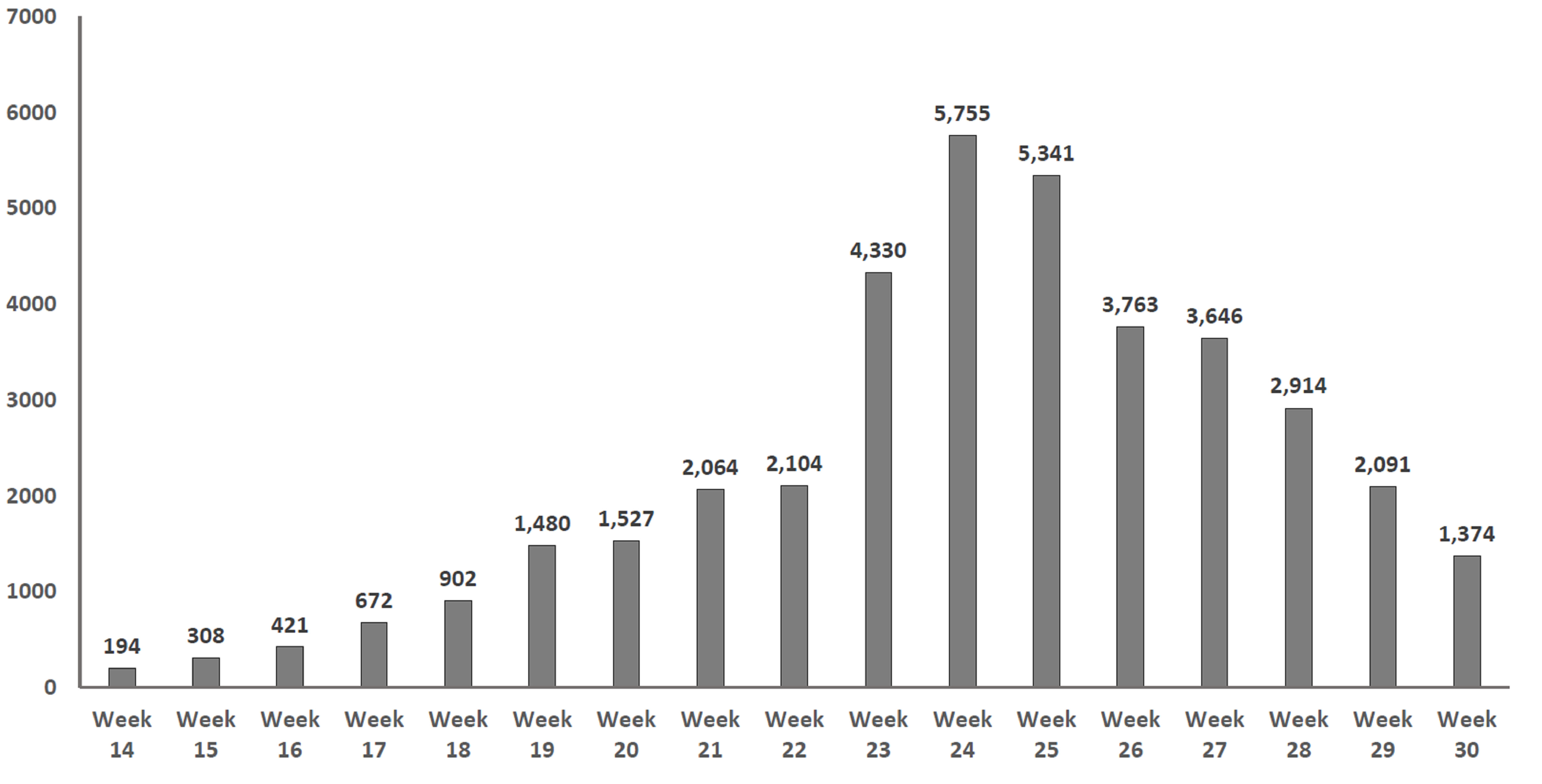

Supplement: S2 Fig — (TIF) [file pone.0249568.s002.tif]
