# Supplementary material for: Detection of SARs-CoV-2 in wastewater using the existing environmental surveillance network: A potential supplementary system for monitoring COVID-19 transmission
Source: PLoS One. 2021 Jun 29;16(6):e0249568. doi: 10.1371/journal.pone.0249568 (PMC8241060; doi:10.1371/journal.pone.0249568)
Supplement: S1 Table — A comparative analysis. (DOCX) [file pone.0249568.s003.docx]

**S1 Table. Wastewater samples collected during March 20, 2020 to April 09, 2020. A comparative analysis.**

| **Sample ID** | **Drainage Type** | **Epi Week** | **District** | **Date Collection** | **Kit 1 (ORF 1+ Ngene)** | **Kit 2 (ORF 1ab+ Ngene)** | **Kit 3  (ORF1ab )** | **Corman V. M. et. al Method (E Gene)** | **Final Results** |
| --- | --- | --- | --- | --- | --- | --- | --- | --- | --- |
|  |  |  |  |  |  |  |  |  |  |
| 186 | PUMPING STATION | Week 12 | RAJANPUR | 20-Mar-20 | ND | ND | ND | ND | ND |
| 189 | OPEN DRAINAGE | Week 12 | QUETTA | 20-Mar-20 | + | + | ND | + | Detected |
| 197 | OPEN DRAINAGE | Week 14 | KOHAT | 30-Mar-20 | ND | ND | ND | ND | ND |
| 198 | OPEN DRAINAGE | Week 14 | KURRAM | 30-Mar-20 | ND | ND | ND | ND | ND |
| 199 | PUMPING STATION | Week 14 | DIKHAN | 30-Mar-20 | ND | ND | ND | ND | ND |
| 200 | OPEN DRAINAGE | Week 14 | DIKHAN | 30-Mar-20 | ND | ND | ND | ND | ND |
| 201 | OPEN DRAINAGE | Week 14 | KAMBAR | 31-Mar-20 | ND | ND | ND | ND | ND |
| 202 | PUMPING STATION | Week 14 | SHEIKHUPURA | 1-Apr-20 | ND | ND | ND | ND | ND |
| 203 | OPEN DRAINAGE | Week 14 | QUETTA | 1-Apr-20 | + | + | + | + | Detected |
| 204 | OPEN DRAINAGE | Week 14 | ABOTABAD | 3-Apr-20 | ND | ND | ND | ND | ND |
| 205 | OPEN DRAINAGE | Week 14 | BANNU | 3-Apr-20 | + | + | + | + | Detected |
| 206 | PUMPING STATION | Week 14 | FAISALABAD | 3-Apr-20 | + | + | ND | + | Detected |
| 207 | OPEN DRAINAGE | Week 14 | SIALKOT | 2-Apr-20 | ND | ND | ND | ND | ND |
| 208 | OPEN DRAINAGE | Week 14 | PISHIN | 2-Apr-20 | ND | ND | ND | ND | ND |
| 209 | OPEN DRAINAGE | Week 14 | KABDULAH | 4-Apr-20 | ND | ND | ND | ND | ND |
| 210 | PUMPING STATION | Week 15 | LAHORE | 6-Apr-20 | ND | ND | ND | ND | ND |
| 211 | PUMPING STATION | Week 15 | DGKHAN | 6-Apr-20 | ND | ND | ND | ND | ND |
| 212 | OPEN DRAINAGE | Week 15 | MIRPUR | 6-Apr-20 | ND | ND | ND | ND | ND |
| ICT-01 | OPEN DRAINAGE | Week 15 | ISLAMABAD | 9-Apr-20 | + | + | + | + | Detected |
| ICT-02 | OPEN DRAINAGE | Week 15 | ISLAMABAD | 9-Apr-20 | + | + | + | + | Detected |

*ND means no viral RNA or less than sensitivity limit.
