# Supplementary material for: Detection of SARs-CoV-2 in wastewater using the existing environmental surveillance network: A potential supplementary system for monitoring COVID-19 transmission
Source: PLoS One. 2021 Jun 29;16(6):e0249568. doi: 10.1371/journal.pone.0249568 (PMC8241060; doi:10.1371/journal.pone.0249568)
Supplement: S2 Table — (DOCX) [file pone.0249568.s004.docx]

**S2 Table.** **Details of wastewater samples tested for SARS-CoV2 at Virology Department, National Institute of Health, Islamabad, Pakistan.**

| **Collection Site** | **Drainage Type** | **District** | **EPI Week** | **COVID-19 Results** |
| --- | --- | --- | --- | --- |
| AQILPUR & ASLAM TOWN | PUMPING STATION | RAJANPUR | Week12 |  |
| SUR PUL | OPEN DRAIN | QUETTA | Week12 | DETECTED |
| FAQIRABAD | OPEN DRAIN | KOHAT | Week14 |  |
| HAZARA COLONY | OPEN DRAIN | KURRAM | Week14 |  |
| COMPSITE BUS STAND & MODC | OPEN DRAIN | DIKHAN | Week14 |  |
| COMPOSITE SHERPAO & ZAFARABAD | OPEN DRAIN | DIKHAN | Week14 |  |
| BAGO ROAD & BAQIRABAD | PUMPING STATION | KAMBAR | Week14 |  |
| QILA SHEIKHUPURA & PS BAHRIAN WALA | PUMPING STATION | SHEIKHUPURA | Week14 |  |
| RAILWAY PUL | OPEN DRAIN | QUETTA | Week14 | DETECTED |
| NALA BHAIR | OPEN DRAIN | SIALKOT | Week14 |  |
| KALAPUL MURRE ROAD | OPEN DRAIN | ABOTABAD | Week14 |  |
| HINJAL & NOORABAD | OPEN DRAIN | BANNU | Week14 |  |
| PUMPING S.36 AHMAD NAGAR | PUMPING STATION | FAISALABAD | Week14 | DETECTED |
| TURWA | OPEN DRAIN | PISHIN | Week14 |  |
| ARMY KAZIBA | OPEN DRAIN | KABDULAH | Week14 |  |
| GULSHAN RAVI STATION | PUMPING STATION | LAHORE | Week15 |  |
| SABZI MANDI | OPEN DRAIN | DG KHAN | Week15 |  |
| SANGOT & THOTHAL | OPEN DRAIN | MIRPUR | Week15 |  |
| WALKWAY NEELUM & OLD NEELUM BRIDGE | OPEN DRAIN | MUZAFARABAD | Week15 |  |
| MIANI PUMPING STATION | PUMPING STATION | SUKKUR | Week15 |  |
| SURAJ MIANI | PUMPING STATION | MULTAN | Week15 |  |
| FRONTIER COLONY | OPEN DRAIN | KARACHI | Week15 | DETECTED |
| ORANGI NALLA | OPEN DRAIN | KARACHI | Week15 |  |
| QASBA COLONY | OPEN DRAIN | KARACHI | Week15 | DETECTED |
| BANGALI PARA | OPEN DRAIN | KARACHI | Week15 |  |
| MUHAMMAD KHAN COLONY | OPEN DRAIN | KARACHI | Week15 |  |
| KORANGI NALLA | OPEN DRAIN | KARACHI | Week15 |  |
| SECTOR I-10/4 | OPEN DRAIN | Islamabad | Week15 | DETECTED |
| SECTOR I-10/1 | OPEN DRAIN | Islamabad | Week15 | DETECTED |
| HIJRAT COLONY PIDC COLONY | OPEN DRAIN | KARACHI | Week15 |  |
| KONRA CHINA & SPAISHTA | OPEN DRAIN | WAZIR-S | Week15 |  |
| SABZI MANDI | OPEN DRAIN | Islamabad | Week16 |  |
| DHOKE DALLAL | OPEN DRAIN | RAWALPINDI | Week16 | DETECTED |
| SAFDAR ABAD | OPEN DRAIN | RAWALPINDI | Week16 | DETECTED |
| MAIN DISPOSAL | PUMPING STATION | DG KHAN | Week16 |  |
| OUTFALL STATION-G | PUMPING STATION | LAHORE | Week16 | DETECTED |
| OUTFALL STATION-H | PUMPING STATION | LAHORE | Week16 |  |
| OUTFALL STATION-F | PUMPING STATION | LAHORE | Week16 |  |
| COMPOSITE SHERPAO & ZAFARABAD | OPEN DRAIN | DIKHAN | Week16 |  |
| COMPSITE BUS STAND & MODC | OPEN DRAIN | DIKHAN | Week16 |  |
| RASHID MINHAS RD LAY | OPEN DRAIN | KARACHI | Week16 |  |
| HAJI MUREED GOTH | OPEN DRAIN | KARACHI | Week16 |  |
| TAWOOS ABAD | OPEN DRAIN | QUETTA | Week16 |  |
| TULSIDAS PUMPING STATION | PUMPING STATION | HYDERABAD | Week16 |  |
| KHAMISO GOTH | OPEN DRAIN | KARACHI | Week16 |  |
| SOHRAB GOTH | OPEN DRAIN | KARACHI | Week16 |  |
| MAKKA PUMPING STATION | PUMPING STATION | SUKKUR | Week16 |  |
| MACHAR COLONY | OPEN DRAIN | KARACHI | Week16 |  |
| BAGO ROAD & BAQIRABAD | PUMPING STATION | KAMBAR | Week16 |  |
| SADDAR PUMPING STATION | PUMPING STATION | JACOBABAD | Week16 |  |
| MASAN MULLAH | PUMPING STATION | DADU | Week16 | DETECTED |
| LANDHI BAKHTAWAR VILLAGE | OPEN DRAIN | KARACHI | Week16 | DETECTED |
| HADI PACKET | OPEN DRAIN | KILLA ABDULLAH | Week16 | DETECTED |
| CHAKORA NULLA | OPEN DRAIN | KARACHI | Week16 |  |
| RAJKOT & SANSI ROAD & PEOPLE COLONY | OPEN DRAIN | GUJARANWALA | Week16 |  |
| GANJ MOHALLA | OPEN DRAIN | ZHOB | Week16 |  |
| CHAK & PAR HOTI | OPEN DRAIN | MARDAN | Week16 |  |
| Rawalpindi Institute of Urology | OPEN DRAIN | RAWALPINDI | Week16 | DETECTED |
| Dhoke Kashmirian | OPEN DRAIN | RAWALPINDI | Week16 | DETECTED |
| PUMPING S.36 AHMAD NAGAR | PUMPING STATION | FAISALABAD | Week16 |  |
| MILL COLONY | OPEN DRAIN | NOWSHERA | Week16 | DETECTED |
| SHAHEEN MUSLIM TOWN | OPEN DRAIN | PESHAWAR | Week16 | DETECTED |
| RASALA LINE | OPEN DRAIN | LORALAI | Week16 |  |
| MULTAN ROAD STATION | PUMPING STATION | LAHORE | Week17 | DETECTED |
| AQILPUR & ASLAM TOWN | PUMPING STATION | RAJANPUR | Week17 |  |
| SUR PUL | OPEN DRAIN | QUETTA | Week17 |  |
| LABOUR NALA | OPEN DRAIN | DERA BUGHTI | Week17 |  |
| KATAN PUL | OPEN DRAIN | KHUZDAR | Week17 |  |
| WAPDA COLONY | OPEN DRAIN | NASIRABAD | Week17 |  |
| ALI TOWN | PUMPING STATION | MULTAN | Week17 |  |
| SILAN WALI | PUMPING STATION | SARGODAHA | Week17 |  |
| LARA MA | OPEN DRAIN | PESHAWAR | Week17 |  |
| KALAPUL MURRE ROAD | OPEN DRAIN | ABOTABAD | Week18 | DETECTED |
| FAQIRABAD | OPEN DRAIN | KOHAT | Week18 | DETECTED |
| SANGOT & THOTHAL | OPEN DRAIN | MIRPUR | Week18 |  |
| LALBAGH & TIBA BAHADUR | PUMPING STATION | BAHAWALPUR | Week18 |  |
| JATAK KILLI & TAKHTHANI | OPEN DRAIN | QUETTA | Week18 | DETECTED |
| KOTLA ABDUL FATAH | PUMPING STATION | MULTAN | Week18 |  |

* Collection Site name is designated as per drainage area or collection vicinity.

*ND (Not Detected) samples mean no viral RNA or viral RNA below the detection limits.
