# Supplementary material for: Detection of SARs-CoV-2 in wastewater using the existing environmental surveillance network: A potential supplementary system for monitoring COVID-19 transmission
Source: PLoS One. 2021 Jun 29;16(6):e0249568. doi: 10.1371/journal.pone.0249568 (PMC8241060; doi:10.1371/journal.pone.0249568)
Supplement: S3 Table — Comparison among the Cq values. (DOCX) [file pone.0249568.s005.docx]

**S3 Table. Sample preparation without and with centrifugation before viral RNA Extraction. Comparison among the *Cq* values.**

| **Lab#** | **Collection Site** | **District** | **Date**  **Collection** | **EPI Week** | **Without Centrifugation step** | | | | | **With Centrifugation step** | | | | |
| --- | --- | --- | --- | --- | --- | --- | --- | --- | --- | --- | --- | --- | --- | --- |
|  |  |  |  |  | **N-GENE** | **Cq** | **ORF-1ab** | **Cq** | **RESULT** | **N-GENE** | **Cq** | **ORF-1ab** | **Cq** | **RESULT** |
| 215 | Frontier Colony | Karachi | 8-Apr-20 | Week15 | + | 37 | + | 38 | DETECTED | + | 35 | + | 37 | DETECTED |
| 217 | Qasba Colony | Karachi | 8-Apr-20 | Week15 | + | 38 | + | 38 | DETECTED | + | 36 | + | 36 | DETECTED |
| 218 | Bangali Para | Karachi | 8-Apr-20 | Week15 | - |  | - |  | NOT DETECTED | - |  | - |  | NOT DETECTED |
| 225 | Dhoke Dallal | Rawalpindi | 13-Apr-20 | Week16 | + | 38 | + | 40 | DETECTED | + | 34 | + | 37 | DETECTED |
| 226 | Safdarabad | Rawalpindi | 13-Apr-20 | Week16 | + | 36 | + | 36 | DETECTED | + | 33 | + | 36 | DETECTED |
| 228 | Outfall Station G | Lahore | 13-Apr-20 | Week16 | + | 38 | + | 39 | DETECTED | + | 29 | + | 31 | DETECTED |
| ICT-04 | Rawalpindi Institute of Urology | Rawalpindi | 17-Apr-20 | Week16 | + | 38 | + | 39 | DETECTED | + | 34 | + | 37 | DETECTED |
| ICT-06 | Dhoke Kashmirian | Rawalpindi | 17-Apr-20 | Week16 | + | 39 | + | 39 | DETECTED | + | 36 | + | 38 | DETECTED |
